# Limitations and Prospects for Diffusion-Weighted MRI of the Prostate

**DOI:** 10.3390/diagnostics6020021

**Published:** 2016-05-27

**Authors:** Roger Bourne, Eleftheria Panagiotaki

**Affiliations:** 1 Discipline of Medical Radiation Sciences, Faculty of Health Sciences, University of Sydney, Sydney, NSW 2006, Australia; 2 Centre for Medical Image Computing, University College London, London WC1E 6BT, UK; e.panagiotaki@cs.ucl.ac.uk

**Keywords:** prostate, cancer, MRI, mpMRI, diffusion, microstructure, modeling

## Abstract

Diffusion-weighted imaging (DWI) is the most effective component of the modern multi-parametric magnetic resonance imaging (mpMRI) scan for prostate pathology. DWI provides the strongest prediction of cancer volume, and the apparent diffusion coefficient (ADC) correlates moderately with Gleason grade. Notwithstanding the demonstrated cancer assessment value of DWI, the standard measurement and signal analysis methods are based on a model of water diffusion dynamics that is well known to be invalid in human tissue. This review describes the biophysical limitations of the DWI component of the current standard mpMRI protocol and the potential for significantly improved cancer assessment performance based on more sophisticated measurement and signal modeling techniques.

## 1. Introduction

Prostate cancer is estimated to account for the deaths of 30,000 Western men annually [1]; however, many forms of prostate cancer are indolent and do not require treatment. Using only traditional assessment techniques it remains unclear and controversial as to which men require aggressive treatment and what form it should take. While the tumour’s grade and volume are the best indicators of malignancy, and thus the need for intervention, at present these can only be reliably measured after surgical removal of the prostate [2,3,4]. Transrectal ultrasound guided (TRUS) biopsy, the standard diagnostic method, has low sensitivity and often underestimates malignancy [5,6,7]. Prostate specific antigen (PSA) screening has increased the rate of prostate cancer detection but resulted in a large number of men with clinically insignificant disease undergoing unnecessary treatment [8,9]. Treating insignificant prostate cancer inflicts otherwise avoidable pain, stress, cost, and complications including impotence and incontinence. There is now a significant discrepancy between the potential of localized therapy and the targeting information available on disease localization. A recent report describes real time motion-compensated delivery of a radiotherapy beam with precision better than 1 mm [10]. Unfortunately, for prostate cancer patients, there is currently no correspondingly precise tumour imaging technique to plan the focal or boosted dose delivery which would maximize treatment effect while causing minimum harm to uninvolved tissues within and adjacent to the prostate.

## 2. Multiparametric Magnetic Resonance Imaging (mpMRI)

### 2.1. mpMRI and Prostate Cancer

Better pretreatment imaging of prostate cancer is critical to reducing unnecessary treatment of insignificant disease while improving the effectiveness and minimizing the harm of interventions. The current best imaging method, multiparametric magnetic resonance imaging (mpMRI), is increasingly being used to target biopsies, assess risk, and select treatment. The mpMRI protocol comprises three independent scans with distinct image contrast mechanisms. Each scan depends on a particular property of tissue water: (1) *T2* on the molecular environment of the water; (2) dynamic contrast enhancement (*DCE*) on blood flow and vessel wall permeability; and (3) *DWI* on tissue microstructure [11,12,13]. Radiologists interpret the T2, DCE, and DWI scans to report on the probable amount and location of any cancer. The PI-RADS^TM^ reporting standard [14] aims to minimize image interpretation subjectivity, and there are UK and European consensus statements relating to standardization of imaging method [15,16].

### 2.2. The Role of Diffusion-Weighted Imaging in mpMRI

The DWI component of the mpMRI scan has far stronger correlations with both cancer grade and volume than T2 and DCE [17,18,19]. This superior performance of DWI relates to the direct dependence of image contrast on differences in the rate of diffusion of water molecules due to tissue microstructure changes. Cancer-associated changes (including the number, size, type, and arrangement of cells) significantly alter the water diffusion behavior. The common categorization of DWI as a ‘functional’ imaging technique is an unfortunate misnomer—no other MRI contrast mechanism is more closely related to tissue structure.

## 3. What is DWI Measuring?

### 3.1. Diffusion-Sensitization of the MRI Signal

The basic DWI method produces a signal that is dependent on the average water molecule displacement over a specific time interval in a direction defined by a pair of ‘diffusion-sensitizing’ magnetic field gradients [20]. Larger average displacements within a voxel (measurement volume element) result in greater DWI signal attenuation. The displacement sensitivity of the DWI measurement can be increased either by increasing the time interval between the diffusion-sensitizing gradients (the ‘*effective diffusion time*’), or by increasing the strength of the gradients. These two independent imaging method parameters are commonly combined and expressed as the diffusion-weighting or ‘*b*-factor’ (or *b*-value).

Depending on the measurement technique, signal attenuation may result from both ‘true’ diffusion (Brownian thermal motion) and flow due to tissue perfusion. The recommended mpMRI protocols [15,16] aim to minimize perfusion effects so that the image contrast relates primarily to the way the tissue microstructure hinders and restricts thermal diffusion. Structurally dense tissue (typical of solid non-necrotic tumours) appear brighter in the diffusion-weighted image than normal tissues with relatively loosely packed cells and an open extracellular matrix. By performing multiple measurements with an array of diffusion-sensitizing gradient orientations the presence and degree of diffusion anisotropy (preferential diffusion in a particular direction) can be quantified. Post-processing of anisotropy data from multiple voxels in a 3D volume enables the mapping and visualization of fiber tracts.

An important but often neglected feature of DWI is the sensitivity to tissue structure on multiple spatial scales. Depending on the time interval of the diffusion-weighted measurement each water molecule is likely to ‘explore’ a larger or smaller range of spatial scales of the tissue microstructure. For long diffusion times structure heterogeneity on the smallest spatial scales will be averaged out and the signal attenuation will depend primarily on large scale tissue structure features. Conversely, at the short time limit there are minimal interactions with the tissue structure and molecular displacement has the Gaussian probability distribution of unhindered Brownian motion. For clinical DWI scans typical diffusion times are around 40–80 ms, corresponding to an unhindered unrestricted mean diffusive water displacement of about 30 μm. By assessing the dependence of signal attenuation on diffusion time it is possible to estimate the dimensions of restricting structures.

The direct dependence of water mobility on the tissue microstructural environment means DWI is sensitive to the arrangement, type, geometry, and permeability of cells at the micron scale—key characteristics that correlate with cancer malignancy.

### 3.2. Signal Models

The inference of specific tissue structure changes from DWI measurements presents a significant and only partially tractable inverse problem. While it is well established that tissue microstructure affects molecular diffusion dynamics, and hence the degree of attenuation of a diffusion-weighted MRI signal, many different tissue structures could potentially lead to the same average water molecule displacement and the same DWI signal. The number of solutions to this inverse problem, and thus the sensitivity and specificity for pathology detection, depends on the information content of the DWI measurement (the data acquisition method) and the ability of the signal analysis model to extract this information (the data processing method).

DWI signal models can be categorized broadly as either phenomenological or structural. Phenomenological models aim for a reliable mathematical description of the DWI signal attenuation as a function of increasing diffusion weighting. Structure-based models predict the DWI signal attenuation based on calculated or simulated diffusion dynamics in one or several structural compartments. The recent historical trend is towards structure-based models as, ideally, the model parameters correspond directly to diagnostic tissue structural features. At present the recommended DWI protocol for prostate mpMRI uses only the very simple phenomenological ‘ADC’ model.

## 4. Limitations of the Standard ‘Apparent Diffusion Coefficient’ (ADC) Model

### 4.1. The ‘Apparent Diffusion Coefficient’ (ADC)

Cancer detection and characterization using clinical DWI methods is currently almost exclusively based on a model that assumes a Gaussian displacement probability for water and a consequent monoexponential decay of the DWI signal (*S*) with increasing diffusion weighting (*b*).

(1) $$S=S_{0}e^{-Db}$$

where *D* is the water self-diffusion coefficient. Because tissue constitutes a highly heterogeneous diffusion environment and the displacement probability is generally non-Gaussian, *D* is conventionally replaced by *ADC*—*an apparent diffusion coefficient.* The use of the indefinite article *‘an’* here is important. The calculated value of ADC may be strongly dependent on both the DWI measurement protocol and the model fitting method, although this is generally not recognized in the radiology literature [21].

The monexponential ADC model is the simplest possible description of DWI signal behavior, and thus a very poor solution to the inverse problem of assessing tissue structure from a DWI measurement. Calculation of a tissue ADC map provides a *semi*-quantitative assessment of gross variations in water diffusion dynamics due to factors that may include cell density, size, shape, permeability, subcellular architecture, extracellular matrix, and perfusion effects. The dependence of ADC on a variety of histological features simultaneously means it lacks biological specificity.

Despite its simplicity the ADC model performs well compared with other MRI contrast methods for prostate cancer detection and grading. There is a moderate negative correlation between ADC and cancer Gleason grade [22,23], and ADC correlates more strongly with histologically determined cancer volume than T2 and DCE parameters [17]. Nevertheless, reported ADC values in prostatic cancer and benign tissue overlap substantially, particularly in the central and transition zones [24,25].

### 4.2. ADC Variations in Prostate Tissue at the Microstructure Scale

Diffusion-weighted magnetic resonance microimaging of formalin fixed prostate tissue has revealed distinctly different diffusion dynamics in the three major gland components. ADC is low in the epithelium, higher in the fibromuscular stroma, and highest in lumen space [26]. Diffusion anisotropy is high in the fibromuscular stroma, and low in the epithelium layer and lumen space [27]. In the stroma the preferential diffusion direction matches the orientation of the smooth muscle cells [27]. Changes in the relative partial volume of these gland components appears to be the most significant contributor to ADC differences detected at the spatial resolution typical of clinical prostate scans (see Section 4.3). Low ADC epithelium has also been found in breast [28] and esophagus [29] tissue; however, the biophysical basis of ADC differences between epithelium and stroma is yet to be elucidated.

### 4.3. ADC and ‘Cellularity’

Cancer-related changes in DWI signal contrast and ADC are conventionally attributed to tissue ‘cellularity’ variations. The commonly reported cellularity metrics are nuclear count per unit area in a histological image, and nuclear area per unit area. While there is substantial evidence for a correlation between these metrics and ADC in neural tissue, there is very limited biophysical evidence that in cancer tissue a higher cell density will, *per se*, result in ADC changes. Besides cell density, membrane permeability and multiple intra- and extracellular structural features will affect the measured ADC. In the prostate, cell type may be more important than cell density. In fixed whole prostates, both ADC and Gleason pattern changes correlate more strongly with relative partial volume of the gland components epithelium, stroma, and lumen than with cellularity metrics [30].

## 5. Improving on ADC—Phenomenological Models

### 5.1. DTI: A Simple Anisotropic Model

Diffusion tensor imaging (DTI) extends the isotropic, monoexponential ADC model to account for diffusion anisotropy. *ADC* is replaced by a 3 × 3 symmetric tensor ***D***. The extra degrees of freedom allow the model to detect anisotropy when there is underlying microstructure with a high degree of structural order. ***D*** summarizes the principal diffusion directions (eigenvectors) and corresponding diffusivities (eigenvalues $\lambda_{1}\geq \lambda_{2}\geq \lambda_{3}$). One of the most commonly used metrics derived from the tensor is the fractional anisotropy (FA) which indicates the degree of any anisotropy:

(2) $$FA=\sqrt{\frac{{\left(\lambda_{1}-\lambda_{2}\right)}^{2}+{\left(\lambda_{1}-\lambda_{3}\right)}^{2}+{\left(\lambda_{2}-\lambda_{3}\right)}^{2}}{2(\lambda_{1}^{2}+\lambda_{2}^{2}+\lambda_{3}^{2})}}$$

Clearly, there are many nonidentical tensors ***D*** that could have identical FA values, emphasizing that FA is a non-specific marker of microstructural order.

At the tissue microstructure scale regions of coherent myocyte orientation in prostate fibromuscular stroma give rise to distinct diffusion anisotropy [27,31]. However, *in vivo* DTI studies of the prostate have yielded inconsistent results for the value of FA in predicting the presence of cancer [32,33,34,35,36,37]. Some of this inconsistency likely results from method variations and the well-known sensitivity of FA to measurement noise [38,39]. Further, at typical clinical imaging spatial resolutions (voxel volume 4–16 mm^3^), incoherent smooth muscle orientation within individual voxels would be expected to reduce the measured FA. A study of whole prostates *ex vivo* demonstrated a consistent and continuous decrease in FA as voxel volume increased, and very large differences in average FA between prostates [40].

Such equivocal results highlight the weakness of the DTI model for describing prostatic tissue, and as a recent study suggests [41], anisotropy may be more informative and reliable as part of a multicompartment model (see Section 7.2).

### 5.2. Higher Order Isotropic Models

The non-monoexponential DWI signal attenuation with increasing *b*-factor has been characterized with a range of mathematical models of variable complexity (see Section 6 for methods of comparing models). The simplest of these assesses the kurtosis of the signal attenuation, or how it deviates from a monoexponential [42]:

(3) $$S=S_{0}e^{-(D_{K}b-\frac{K}{6}D_{K}^{2}b^{2})}$$

In this model the ADC is replaced by a kurtosis-adjusted diffusivity ($D_{K}$). The kurtosis (*K*) has no biophysical interpretation, but has been reported to provide more accurate cancer detection (higher area under an ROC curve) than ADC [43,44].

For DWI measurements that include very high diffusion weightings a biexponential model is preferred:

(4) $$S=S_{1}e^{-D_{1}b}+S_{2}e^{-D_{2}b}$$

This is basically a ‘two ADCs’ model and can be interpreted to represent two distinct pools of water, though the temptation to assign the pools to specific tissue compartments should be resisted [45]. The biexponential is the basis of the ‘IVIM’ (intravascular incoherent motion) model [46] that can be used for measurements that include intermediate and very low diffusion weightings and thus may be significantly affected by tissue perfusion. In this instance the very high ADC of one of the water components enables its unequivocal assignment to blood flow rather than thermal diffusion. The IVIM model has shown inconsistent results for prostate cancer assessment. While some studies found poor diagnostic value compared to ADC [47,48,49], others report significant differences between the IVIM parameters for benign and cancer tissue [50,51]. Perhaps surprisingly, there appears to be no consistent agreement between the characteristics of the perfusion attenuated signal and dynamic contrast enhancement (DCE) model parameters. One study reported lower perfusion fractions in cancer tissue [49], contrary to DCE-MRI studies and expected tumour angiogenesis, while another found opposite correlations [52].

The stretched exponential model assumes that tissue microstructure heterogeneity results in the the presence of a large number of Gaussian components each with different diffusivities [53]:

(5) $$S=S_{0}e^{-{\left(D_{s}b\right)}^{\alpha }}$$

where *α* is the ‘stretching factor’ and $D_{s}$ is a ‘stretch-adjusted’ diffusivity.

Phenomenological models permit only limited physical interpretation, however, by comparing the theoretical information content of different signal models applied to the same measurement data (see Section 6.2) it is possible to make general inferences about underlying tissue structure.

## 6. Model Selection and Performance Testing

Phenomenological models of prostate DWI have generally been compared either by assessment of their cancer prediction performance or by estimation of their relative theoretical information content.

### 6.1. Correlation of Model Parameters and Tissue Pathology

A major weakness of almost all of the studies that compare models on the basis of cancer prediction performance is that the common practice is to correlate individual parameters of multi-parameter models with pathology. In this instance only a fraction of the model’s information content is being tested and the implicit purpose of using a higher order model may be defeated, except in the fortuitous and unpredictable circumstance when a particular parameter contains most of the diagnostic information. Only very recently have a few authors begun to assess the predictive value of parameter combinations and thus incorporate all model information [54].

### 6.2. Model Ranking Based on Information Theory

As an alternative to pathology detection performance, especially in the absence of large sample sizes and reliable correlation of imaging and histopathology ‘truth’ data, competing DWI data models can be compared in terms of their theoretical information content. The implicit assumption of this approach is that a higher information content implies greater microstructure prediction accuracy *from a specific set of measurement data* (see Section 6.3).

A highly parameterized model will in general always fit a given data set more closely (‘better’) than a model with fewer parameters. But do the extra model parameters have any physical meaning in terms of the measured system? Applied to the problem of model selection from inevitably noisy measurement data, information theory aims to balance the inherent parameter bias of overly simplistic models against ‘overfitting’ and consequent high parameter variance of highly parameterized models [55]. The Akaike Information Criterion (AIC) [56], and similar Baysian Information Criterion [57], provide an estimate of the *relative* distance of competing models from an unknowable ‘truth’. In the specific case of water diffusion in biological tissue the truth is immensely complex and the best we can aim for is a diagnostically informative approximation.

Applied to prostate DWI, the information theory approach demonstrates that in whole organs examined *ex vivo* (eliminating perfusion effects) with a wide range of diffusion weightings the 3-parameter biexponential model has a significantly higher information content than 1-parameter ADC, and 2-parameter kurtosis and stretched exponential models [45]. This result enables the general inference that there exist *two* water diffusion environments with distinct ‘structure densities’ leading to two different apparent diffusion coefficients. The comparatively low information content of the ADC, kurtosis, and stretched exponential models indicates that there is not simply one heterogeneous structural environment leading to a continuum of diffusion coefficients. *In vivo* prostate DWI studies support the existence of two diffusion environments distinct from the vasculature [58,59]. Application of the stretched exponential model to the individual components of the biexponential model demonstrates the two distinct diffusion environments are internally heterogeneous [60], but further basic science studies are required to identify these environments histologically. These results, while providing some insight into the complexity of water diffusion dynamics in prostate tissue, highlight the current absence of a clear biophysical understanding of the mechanisms of a powerful clinical imaging technique.

### 6.3. The Importance of Imaging Method

Whatever approach is used for model selection it is important to note that the imaging protocol is critical. With a given set of measurement data the best model is *conceptually* the one that extracts most information from the inevitably noisy data, and *practically* the one that provides the most accurate (sensitive and specific) pathology prediction. If the imaging protocol is inappropriate then the measurement data will not contain information that predicts pathology. Improving the clinical performance of DWI thus depends on optimization of both the measurement technique and the signal analysis model. Because of the inherently low signal-to-noise ratio of MRI in general, and DWI in particular, there are significant technical, practical, and financial constraints on obtaining an information-rich measurement.

For prostate DWI, imaging method considerations appear in a plethora of research publications around the theme of an ‘optimum *b*-factor’ for cancer detection. Unfortunately, as mentioned in Section 3.1, the importance of diffusion time in defining the structure-scale sensitivity of the imaging method is generally not recognized and diffusion times are very rarely reported. Different MRI scanners are likely to generate the same *b*-factor according to the available maximum gradient strength (which may vary widely between scanners) and thus using very different diffusion times [21]. As yet, the diffusion time-dependence of ADC measurements in prostate tissue has not been investigated.

### 6.4. Model-Based Image Synthesis

Because of reports that acquisitions performed with higher *b*-factors have greater cancer detection accuracy there has been strong recent interest in methods that compute a high *b*-factor image using only low and intermediate *b*-factor scans [61]. This ‘computed high *b*-value DWI’ uses a diffusion model (most commonly ADC, but kurtosis and IVIM have been used) to produce high contrast diffusion-weighted images with lower noise than images actually acquired with the high *b*-factor. There are variable reports of diagnostic value [62,63], and again, possible effects of diffusion time differences have been neglected. Computation of a high *b*-factor image should be regarded as a noise reduction technique since the computed image contains no information that is not present in the actually acquired low and intermediate *b*-factor scans. *‘Apparent high b-value DWI’* would be more appropriate term for this model-based technique [64].

## 7. Compartment Models

### 7.1. Two-Compartment Models

Compartment models aim to make a direct assignment of the microstructural features that cause signal changes. Ideally the model components will relate closely to diagnostic tissue features. A common approach uses compartment models that describe the DWI signal as the sum of separate signals arising from separate (non-mixing) populations of water molecules in distinct structural environments. Most previous studies have quantified properties of neural tissue [65,66], but recent applications of structural models to the diffusion signals of non-neural tissue show promise.

The intravoxel incoherent motion (IVIM) model (see Section 5.2) separates the signals from vascular and non-vascular water, however, its description of diffusion in the cellular component of the tissue does not account for the known presence of two clearly distinct “slow” and “fast” diffusion environments. This deficiency of the IVIM model may be responsible for inconsistent estimates of the fast and slow diffusion parameters [49,52,67].

### 7.2. A Three-Compartment Model: VERDICT

The recent VERDICT (Vascular, Extracellular, and Restricted Diffusion for Cytometry in Tumours) framework addresses some of the limitations of the IVIM model by using two isotropic components to describe the non-vascular diffusion environment [68]. Significantly, VERDICT addresses the diffusion time dependence of the signal attenuation by modeling the intracellular space as an impermeable spherical pore. For *in vivo* data the inherently low signal-to-noise ratio necessitates model fitting based on fixed diffusion coefficients and the model provides estimates of $f_{\mathrm{EES}}$ (extracellular/extravascular space volume fraction), $f_{\mathrm{IC}}$ (intracellular (IC) volume fraction), $f_{\mathrm{VASC}}$ (vascular volume fraction), and *R* (cell radius). $f_{\mathrm{IC}}/R^{3}$ provides a measure of cell density.

A preclinical application of VERDICT in a colorectal cancer model [68] provided estimates of cancer cell size (*R*), cell density ($f_{\mathrm{IC}}/R^{3}$), and vascular volume ($f_{\mathrm{VASC}}$) in close agreement with histology. More importantly, these parameters distinguished tumour cell lines and detected the effects of chemotherapy, while the standard ADC and IVIM techniques failed. An *in vivo* pilot of prostate cancer showed qualitatively good discrimination of cancer and benign tissue [58], however, this study used long imaging protocols which are not feasible for routine clinical use. More recently an accelerated VERDICT protocol for prostate [69] was developed using an optimization technique [70] to identify a feasible measurement protocol that accommodates clinical and hardware constraints.

One of the current limitations of VERDICT is the neglect of diffusion anisotropy in the prostate. A possible reason for inconsistent results from DTI-based estimates of diffusion anisotropy (see Section 5.1), separate from the highly heterogeneous stromal fiber orientation, is the masking of sub-voxel diffusion anisotropy by the presence of a significant ‘background’ pool of water having isotropic diffusion dynamics. This possibility has been addressed in a study of whole prostates *ex vivo* that compared ten different compartment models [41]. This study found that under all measurement conditions tested the highest information content models were those that included both an isotropic restricted diffusion component *and* an unrestricted anisotropic component. Fractional anisotropy (FA) calculated from the anisotropic component of the two-component models was higher than FA calculated from the DTI model—indicating the presence of a masking effect. It remains to be determined whether this more sensitive anisotropy detection method has any diagnostic value.

The *ex vivo* prostate study [41] supported the generality and broad applicability of VERDICT in several ways:

- Model selection results were largely independent of voxel size—indicating that the successful modeling of ‘true’ diffusion in the non-vascular space as one restricted and one unrestricted compartment is not strongly dependent on the amount of subvoxel structure heterogeneity.
- Model selection results were largely independent of maximum *b*-factor.
- The diffusivity parameters were not fixed during model fitting in the *ex vivo* study, but still returned average values similar to the fixed diffusivities used in fitting VERDICT to the relatively noisy *in vivo* data. This provides an independent validation of the fixed values used for the *in vivo* data fitting.

## 8. Future Directions

The role of DWI in prostate pathology assessment is in a very early stage of development and although demonstrably effective, the current standard ADC model could not be less sophisticated. More advanced signal analysis models show promise of improved accuracy but in many cases have not been appropriately tested and the DWI acquisition methods are inconsistent despite attempts to implement a consensus-based standard. Incomplete reporting of critical measurement parameters may be resulting in an underestimation of the diagnostic accuracy of DWI methods. Recent basic science investigations have improved our understanding of the way prostate tissue microstructure determines the nature of contrast in diffusion-weighted images, but significant issues remain. The priorities for prostate DWI method development should address:

**Diffusion time.** The diffusion time dependence of DWI measurements needs to be clarified and diffusion time reported in all published studies to enable controlled meta-analysis. The consensus methods should include a specification of recommended diffusion time. At present only the VERDICT method specifically accounts for and exploits the diffusion time dependence of the signal.
**Membrane permeability.** At present, the multi-compartment structural models and multi-component phenomenological models assume no exchange of water between the compartments/components during the DWI measurement. Studies of a range of cell types in suspension found that membrane permeability alterations produced significant effects on DWI model parameters [71,72]. Although technically challenging, incorporation of water exchange may be an important component of DWI model optimization for clinical applications.
**T2 relaxation.** Current multi-component models also neglect or implement strategies to minimize potential complications due to the possible presence of multiple water pools with different spin-spin (T2) relaxation rates, despite evidence of their existence in prostate tissue [73,74,75]. There are, as yet, no studies that investigate whether the two main water pools identified in diffusion analyses have a direct one-to-one correspondence with the apparently distinct T2 water pools.
**Diagnostic accuracy.** The complex diffusion dynamics of biological tissue means that appropriately developed multi-component models are likely to supersede the current ADC method used in prostate mpMRI. It is essential that assessment of the clinical performance of these models is based on testing of their total information content by using methods that correlate pathology and tissue structure features with the combined model parameters.

## Abbreviations

The following abbreviations are used in this manuscript:

MRI: Magnetic resonance imaging
DWI: Diffusion-weighted magnetic resonance imaging
ADC: Apparent diffusion coefficient
DTI: Diffusion tensor imaging
FA: Fractional anisotropy
VERDICT: Vascular, Extracellular, and Restricted Diffusion for Cytometry in Tumours
